# Improving the Electrochemical Stability of a Polyester–Polycarbonate Solid Polymer Electrolyte by Zwitterionic Additives

**DOI:** 10.1021/acsaem.2c01641

**Published:** 2022-07-19

**Authors:** Isabell L. Johansson, Christofer Sångeland, Tamao Uemiya, Fumito Iwasaki, Masahiro Yoshizawa-Fujita, Daniel Brandell, Jonas Mindemark

**Affiliations:** † Department of Chemistry−Ångström Laboratory, Uppsala University, Box 538, SE-751 21 Uppsala, Sweden; ‡ Department of Materials and Life Sciences, Sophia University, 7-1 Kioi-cho, Chiyoda-ku, Tokyo 102-8554, Japan

**Keywords:** polymer electrolytes, zwitterion, additive, cycling stability, NMC, lithium batteries

## Abstract

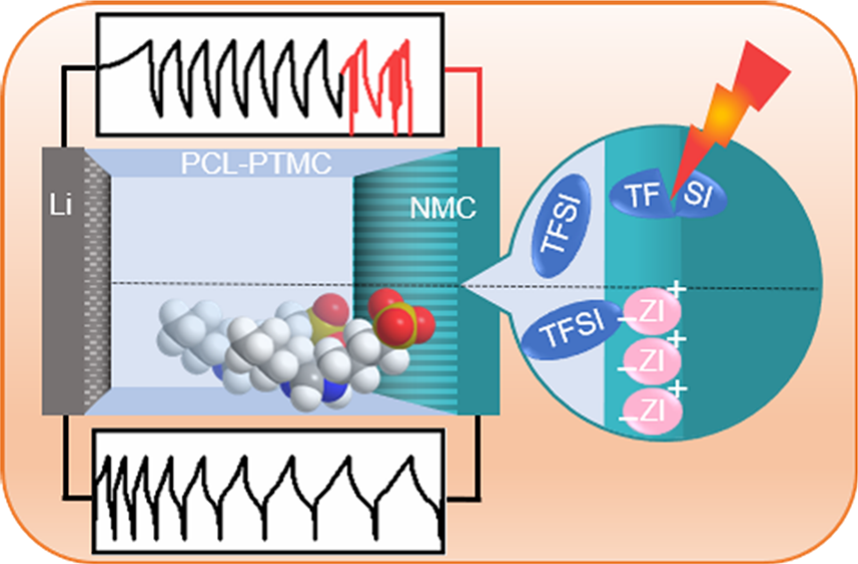

Rechargeable batteries with solid polymer electrolytes (SPEs), Li-metal anodes, and high-voltage cathodes like LiNi_*x*_Mn_*y*_Co_*z*_O_2_ (NMC) are promising next-generation high-energy-density storage solutions. However, these types of cells typically experience rapid failure during galvanostatic cycling, visible as an incoherent voltage noise during charging. Herein, two imidazolium-based zwitterions, with varied sulfonate-bearing chain length, are added to a poly(ε-caprolactone-*co*-trimethylene carbonate):LiTFSI electrolyte as cycling-enhancing additives to study their effect on the electrochemical stability of the electrolyte and the cycling performance of half-cells with NMC cathodes. The oxidative stability is studied with two different voltammetric methods using cells with inert working electrodes: the commonly used cyclic voltammetry and staircase voltammetry. The specific effects of the NMC cathode on the electrolyte stability is moreover investigated with cutoff increase cell cycling (CICC) to study the chemical and electrochemical compatibility between the active material and the SPE. Zwitterionic additives proved to enhance the electrochemical stability of the SPE and to facilitate improved galvanostatic cycling stability in half-cells with NMC by preventing the decomposition of LiTFSI at the polymer–cathode interface, as indicated by X-ray photoelectron spectroscopy (XPS).

## Introduction

Lithium metal batteries containing solid polymer electrolytes (SPEs), entirely free of any type of low-M_w_ solvent, have become an important branch of electrolyte research in the battery field. This type of materials is of interest because SPEs offer a solution to issues like electrolyte flammability, electrolyte leakage, and formation of dendrites in softer electrolytes that results in excessive electrolyte degradation.^1−4^ While the threat of lithium dendrites is present when using lithium metal as anode, replacing the conventional graphite anode with lithium metal is still of interest because it operates at a low standard electrochemical redox potential (−3.04 V *vs* SHE) and lithium metal offers a higher capacity. Being able to combine these properties of the Li-metal anode with a high-voltage cathode, *e.g.,* LiNi_*x*_Mn_*y*_Co_*z*_O_2_ (NMC), is a vital step to meet the energy density needs of our future society.

SPEs are commonly based on poly(ethylene oxide) (PEO), but due to semicrystallinity,^5^ low transference number,^6−8^ and limited electrochemical stability,^5^ other polymeric hosts have been explored as possible alternatives to PEO in recent years.^9^ One such material is poly(ε-caprolactone-*co*-trimethylene carbonate) (PCL-PTMC). This copolymer has shown to be a suitable substitute for PEO because it is an excellent host to lithium ions^10^ and sodium ions,^11^ shows high transference number and ion conduction properties,^12,13^ is fully amorphous, and shows good cycling properties at room temperature.^12^ Being fully amorphous, the PCL-PTMC host can also be used to emphasize the function of certain additives. When used in PEO or PCL, additives often also improve properties such as ionic conductivity by reducing the crystallinity of the host material, making it difficult to separate the function and benefits of using the additive from the effects of reduced crystallinity in the host material.^14,15^

It does, however, require some fine-tuning to reach its full potential. Like any typical SPE host, PCL-PTMC has poor mechanical stability at high temperatures, which is correlated to the increase in segmental motion in polymers with temperature. This has previously been resolved by various kinds of cross-linking;^16,17^ but while mechanical stabilization was shown to be possible through the use of such additives, it at the same time lowered the electrochemical stability of the SPE, which is another property of PCL-PTMC that should be improved to realize cycling against high-voltage cathodes.

Despite the promising potential of polymer electrolytes, cycling versus high-voltage cathodes remains challenging. So far, while cycling with LiFePO_4_ (LFP) is unproblematic for most materials, stable cycling against NMC is often unsuccessful with SPEs.^17−21^ This is true for PEO and likely also for PCL-PTMC. In commercial liquid electrolytes, such instability issues are often resolved with additives that form passivating films on the cathode or increase the electrochemical stability of the system in other ways. Finding additives that can accomplish the same in SPEs—without compromising the conductivity or mechanical stability—would be highly desirable.

Zwitterions as additives in liquid electrolytes^22,23^ and polymer electrolytes, such as those based on PEO and PEGDME,^24−27^ have shown to improve the electrochemical stability and cycling performance against LiCoO_2_.^28^ The reason for the stability-enhancing effect of zwitterions has so far been explained by improved Li-ion diffusion, as indicated by pulsed-field-gradient NMR in ionic liquids;^29^ by assisting in the formation of a passivating layer;^22,24−26,29,30^ or by the zwitterionic additives interacting with the solvent to decrease the reactivity in liquid electrolytes.^24,31^

Characterizing the electrochemical stability in solid polymer electrolytes is notoriously difficult.^32^ Changing one experimental parameter, such as the potential sweep rate or working electrode, can give widely different results for the same electrolyte; one example is how the scan rate during a cyclic voltammetry experiment can determine the observed onset of electrochemical degradation. The true reason why most SPEs are not able to cycle against high-voltage cathodes has so far not been properly explored, and the voltammetric methods employed to determine the electrochemical stability of SPEs do not provide answers that are comprehensive enough to resolve these types questions. Despite the often wide electrochemical stability window indicated by techniques like linear sweep voltammetry (LSV) and cyclic voltammetry (CV), batteries fail during cycling due to the inferior stability of the SPE in the real battery cell setup. If the conventional techniques employed to characterize the electrochemical stability are not sufficient at determining this, other methods need to be utilized as replacements or as supplements.

In this work, investigations of the origin of the incompatibility between SPEs and high-voltage cathodes were performed. Zwitterions were used as cycling stability-enhancing additives to explore if the electrochemical stability, as determined by voltammetric methods, of the PCL-PTMC copolymer can be increased enough to allow cycling against NMC or, if this is not the case, to give insights to what the associated problem might be. The voltammetric methods were used in conjunction with a method named cutoff increase cell cycling, or CICC, presented previously,^32^ which evaluates the electrochemical performance of a battery cell during cycling with the relevant electrode materials. Finally, the effect of the zwitterionic additives on the interface of cycled cells was investigated with XPS.

## Experimental Section

### Materials

Poly(ε-caprolactone-*co*-trimethylene carbonate) (PCL-PTMC) was synthesized according to published procedures.^10,33^ Poly(ε-caprolactone) (Perstorp, Capa 6500) and acetonitrile (anhydrous, 99.8%, Sigma-Aldrich) were used as received. Lithium bis(trifluoromethylsulfonyl)imide (LiTFSI) (BASF) was dried at 120 °C for 48 h in a vacuum before use. Bim3S and Bim4S were synthesized according to previous reports.^34−36^ Their molecular structures were confirmed by ^1^H NMR. NMC-111 electrodes were prepared in an Ar atmosphere by mixing a slurry of 75 wt % NMC-111 (CustomCells), 10 wt % carbon black (Imerys, C-NERGY SUPER C65), and 15 wt % PCL-PTMC as binder in acetonitrile. A doctor blade was used to coat the slurry on a carbon-coated aluminum foil. After drying overnight in a vacuum, the electrodes were cut to 12 mm discs and dried at 120 °C for 5 h in a vacuum before use. Commercial NMC-111 (CustomCells, 2.0 mAh/cm^2^) was also utilized for some measurements; these were cut as 13 mm discs and dried at 120 °C under a vacuum for 12 h before use.

### Electrolyte Film Preparation

The preparation of solid polymer electrolyte films was done through solvent casting according to experimental details of a previous publication,^10^ with a salt concentration of 20 wt % LiTFSI, based on the total mass of the solid components (not the solvent). Handling and storage of all chemicals, and all cell assembly steps, were performed inside an Ar-filled glove box.

### Fourier-Transform Infrared Spectroscopy (FTIR)

FTIR spectra were recorded using a PerkinElmer Spectrum 100 FT-IR spectrometer with a MIR TGS detector in ATR mode with a Golder Gate ATR (Specac). Spectra were acquired at 25 °C by measuring 32 scans in the wavenumber range 4000–600 cm^–1^ with a resolution of 4 cm^–1^.

### Thermal Properties

The thermal properties of the electrolytes were evaluated by differential scanning calorimetry on a TA Instruments Q2000. Samples were hermetically sealed in Al pans, and the heat flow was measured between 80 and −80 °C in a heat–cool–heat cycle at a heating rate of 10 °C/min and cooling rate of 5 °C/min. The glass transition temperature, *T*_g_, was taken at the midpoint of the heat capacity change.

### Ionic Conductivity

The ionic conductivity was measured using a Schlumberger SI 1260 Impedance/Gain-phase Analyzer instrument on SPEs sandwiched between two stainless steel blocking electrodes, at an AC amplitude of 10 mV over a frequency range of 1 Hz to 10 MHz, from room temperature up to 100 °C. The cells were prepared as coin cells and were annealed at 100 °C for 1 h the day before measurement to improve interfacial contact.

### Electrochemical Characterization

Cyclic voltammetry (CV) was performed on a Bio-Logic VMP2 at 40 °C, between 3.0 and 5.0 V, at a scan rate of 0.1 mV/s. Lithium metal was used as the counter and reference electrode, with the stainless steel of the coin cell as the working electrode. The same type of cell setup was used for staircase voltammetry (SV), where the current response was measured in the same potential range and at the same temperature as for the CV measurements; the potential was increased at steps of 100 mV and held at that potential for 1 h, followed by a 1 h pause before proceeding to the next potential increase. For the PCL cells, the measurements were performed on a Bio-Logic SP240 at 80 °C to improve the current response.

Galvanostatic cycling using the intermittent current interruption (ICI) method,^37^ where the current was interrupted for 1 s at 5 min intervals, with cutoff increase cell cycling (CICC) was carried out on half-cells with Li-foil as anode (15 mm in diameter) and an NMC-111 cathode (12 mm in diameter) placed on top of the SPE. The cells were cycled at a rate of 0.025 mA cm^–2^, which roughly corresponded to a C-rate of C/20. The lower cutoff was 3.0 V, and the upper cutoff was 4.2–5.0 V, with five cycles at each cutoff potential. During cycling, the cells were kept in an oven at 40 °C.

### X-ray Photoelectron Spectroscopy

The XPS measurements were performed on a PHI 5500 instrument with monochromatized Al Kα radiation (1486.6 eV). To facilitate battery disassembly for the measurements, high-molecular-weight (HMW) PCL was utilized as the electrolyte host material, and a commercial NMC-111 electrode was used. The cells were charged and discharged five times at 40 °C before measurement. Due to the low ionic conductivity and low transference number of the HMW PCL, the cycling rate was performed with a current of 0.001 mA cm^–2^, between 4.5 and 3.0 V, for five full charge–discharge cycles.

Postmortem analysis of the cells was performed on the PCL:LiTFSI solid electrolyte layer adjacent to NMC-111, with energy calibration done according to LiTFSI peaks in the S2p spectra. The neutralizer was set on 20 mA and 20%.

## Results and Discussion

**Figure 1:**
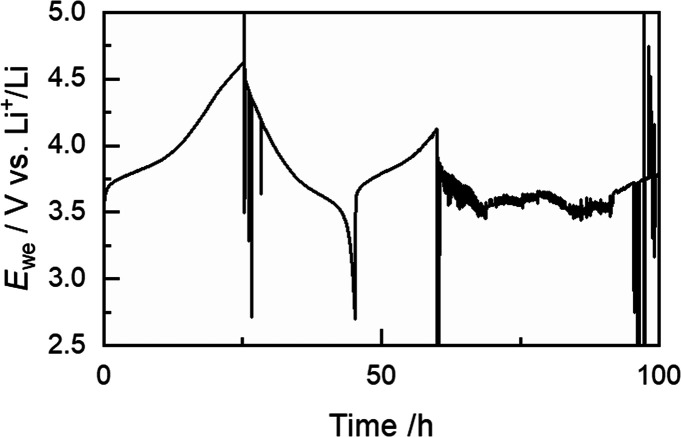
Example of cycling of a Li|PCL-PTMC:LiTFSI|NMC-111 cell with the current density 0.025 mA cm^–2^. During the first cycle, the cell shows signs of short-circuit, and by the second cycle, the ″arbitrary voltage-noise″ behavior manifests.

When LFP is used as cathode material, galvanostatic cycling with the PCL-PTMC:LiTFSI SPE is possible even at room temperature.^12^ However, when switching to a higher-voltage cathode, such as NMC-111, the common cycling behavior is an exceptionally long first charging cycle or failure within a small number of cycles, as is exemplified in Figure 1. This example is just one of many cells with this specific composition, but it is also the only one that managed to complete a full cycle. The arbitrary voltage noise behavior, which is visible in the cell’s second cycle, is a common behavior for these cells during the first charging and is indicative of unwanted side reactions. Despite a number of outstanding qualities in the PCL-PTMC system, being able to consistently cycle against a high-voltage cathode is one of the milestones that needs to be reached with this polymer system, as it likewise is for the more conventional PEO.^4,38^

To improve the cycling performance and the electrochemical stability of PCL-PTMC, electrolyte additives were utilized in this present study. When studying the effect of additives, using a semicrystalline polymer material is unsuitable because additives often affect the crystallinity of such materials. The analysis would then be complicated as any improvements could be due to the effect of the additive, a reduction of crystallinity due to the additive, or both at the same time. By using the fully amorphous host material PCL-PTMC, any effects due to polymer crystallinity will be avoided. Additionally, the PCL-PTMC host has shown long-term stable cycling with LFP at room temperature^12^ and at an elevated temperature when the host material is mechanically stabilized through various cross-linking methods.^16,17^

**Figure 2:**
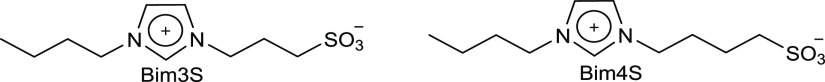
Molecular structures of the zwitterions Bim3S and Bim4S.

Previously, zwitterions have demonstrated the ability to improve the cycling properties of other electrolyte systems when added in modest amounts. The mechanism behind the enhancement is proposed to be due to the zwitterion’s effect on the interface between the polymer and electrode. Possible mechanisms are (1) the prevention of salt anions from approaching the electrode surface and (2) the formation of a passivating film on the electrode surface.^39^ Two zwitterions with varied alkyl spacer length, Bim3S and Bim4S,^35,36^ shown in Figure 2, were investigated as additives in PCL-PTMC electrolytes. These have previously been examined in other polymer systems.^24,25,28^ The Bim4S zwitterion is expected to have a higher flexibility than the Bim3S zwitterion due to the length of the sulfonate-bearing side chain on the molecule. This difference in structure affects their mobility and solubility; at least 1.5 wt % Bim3S and up to 5 wt % Bim4S could be dissolved (see Figure S1) in PCL-PTMC:LiTFSI; to the naked eye, there appeared to be no obvious mechanical or visual difference between the electrolytes with or without zwitterions.

### Thermal Properties and Ionic Conductivity

**Figure 3:**
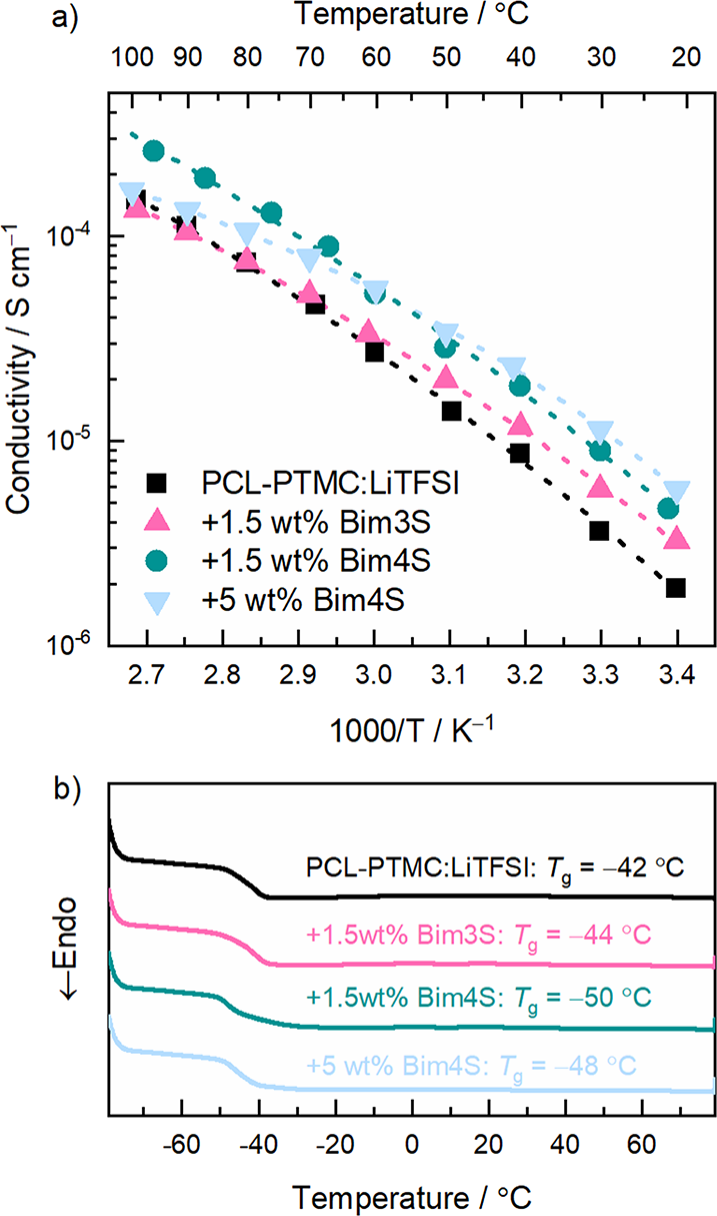
(a) Ionic conductivity as a function of temperature with Vogel–Fulcher–Tammann (VFT) fitting shown as dotted lines and (b) DSC traces of PCL-PTMC:LiTFSI; the *T*_g_ values are obtained at the inflection point.

The ionic conductivity and the thermal properties in PCL-PTMC:LiTFSI with and without zwitterionic additives were measured by EIS and DSC, respectively (see Figure 3). The conductivity data were fitted to the Vogel–Fulcher–Tammann equation (fitting parameters are shown in Table S1) to show the temperature dependence of the ionic conductivities of these materials. A slight reduction in glass transition temperature was seen when the Bim4S zwitterion was added; this was also reflected in a slight increase in ionic conductivity seen in the impedance measurements. The samples containing Bim4S showed the lowest *T*_g_ as well as the highest ionic conductivity. The plasticizing effect and increased ionic conductivity are likely due to the higher molecular flexibility in Bim4S compared to Bim3S, but it cannot be ruled out that these zwitterions function as ″dissociation enhancers″, *i.e.*, increasing the dissociation of the Li-salt, due to the inherently high dipole moment of a zwitterionic molecule wherein the positive and negative charges are closely tethered, as is suggested in a previous study highlighting the effect of Bim4S in an acrylate-based copolymer system.^35^ The sample containing 5 wt % Bim4S has a deviating behavior compared to the other samples, showing a higher ionic conductivity at room temperature than the other compositions but a lower ionic conductivity at the highest temperature. A possible explanation for this can be found in the parameters of the VFT equation (Table S1**)** where the *B*/*T*_0_ term describes the fragility, or the degree of decoupling, of the system.^40^ However, without more samples and data points, it is not possible to assuredly draw firm conclusions from these parameters, and for this reason, further discussion will not be dealt with in this paper. Furthermore, FTIR spectra of PCL-PTMC:LiTFSI with and without zwitterions were recorded (see Figure S2), showing a change in the interaction between the carbonate and ester groups with the Li^+^ but no apparent change in the 700 cm^–1^ region, which can be used to probe changes in the ion coordination.^41^

### Voltammetric Measurements

The electrochemical stability of an electrolyte is conventionally evaluated with voltammetric measurements. An increase in the current generated during the potential sweep is an indication that the electrolyte is unstable, and a reduction or variation in current response of a modified electrolyte is therefore considered a sign that the electrochemical stability is increased. If the electrochemical stability is improved within the potential window relevant for NMC when zwitterions are added by any of the previously proposed mechanisms through interactions with the electrolyte salt or solvent, the electrolytes are expected to also be able to show a more stable cycling performance with NMC.

In the field of electrolytes, the most commonly employed methods to investigate the electrochemical stability of a system are sweep voltammetry, *i.e.*, CV and LSV. For liquid electrolytes, in which the kinetics are sufficiently fast, sweep voltammetry can give valuable information about the complex behavior of reversible reactions. When attempting to apply these experimental techniques on SPEs, some adaptations have to be made for the experimental setup and the analysis of the data, such as greatly reducing the scan rate to make up for the slow kinetics of the polymer matrix. For the investigation of irreversible reactions, such as electrochemical degradation, additional precautions also need to be taken.^32^ Several research groups have published different ways to quantify the electrochemical stability of SPEs, with varied methods to address bias and subjectivity when interpreting data from CV and LSV measurements.^42−44^ In a previous study within our group, we have suggested and tested several experimental methods for investigating the electrochemical stability of SPEs, which are better suited for these types of materials and thereby to analyze the limitations of the solid-state electrolytes.^32^

In this work, CV measurements are included in the Supporting Information (see Figures S3 and S4). Obvious difficulties appeared for interpreting the results, *e.g.*, determining if the maximum current reached was more indicative of the electrochemical (in)stability or if rather the decrease in current between the first cycle and the following cycles was more telling of this property. In the second case, the decrease in current between cycles could be indicative of a passivating layer forming during the first cycle. It was thereby not immediately clear which SPE composition was the most stable, and deciding which behavior (between Bim3S and Bim4S) was an indication of enhanced electrochemical stability becomes highly arbitrary if relying on those methods. In the first cycle, the PCL-PTMC:LiTFSI sample displayed a minor current response after 4.0 V, with a more rapid increase after 4.5 V (more easily seen in Figure S4), while this reference sample without zwitterions showed a more reduced current response in subsequent cycles. The lowest current response during the first cycle was shown for the sample with 1.5 wt % Bim3S; additionally, the current response was also almost constant for this sample during all five cycles. However, since the other samples displayed a decreasing trend in current response during cycling, by the third and fourth cycles, this composition displayed the highest current. It could thus just as well be argued that it had the poorest electrochemical stability among all investigated compositions.

Aside from this, it is important to note in the CVs that the reference sample does not show an exponential response in currents below 4.5 V. In the cycles following the first, the reference sample even has a fairly low current generated at 5.0 V; this would typically be interpreted as the sample being electrochemically stable. Generally, battery cells containing a PCL-PTMC:LiTFSI electrolyte and NMC electrodes show cell failure around 4.2–4.5 V, where the current response in Figure S3 still is relatively low. It can therefore be concluded, as has been previously shown,^32^ that CV is not an appropriate tool for accurately evaluating the electrochemical stability of SPEs to be used with high-voltage cathodes such as NMC.

The samples in Figure S3 containing Bim4S generate a higher current during the CV measurements than the sample containing Bim3S, with a higher amplitude in current for the sample with 5 wt % compared to 1.5 wt % Bim4S during the first cycle. Following the hypothesis that the zwitterions form a passivating film on the electrode surface, it would make sense that the sample with 5 wt % Bim4S has a higher current response since there are more zwitterions available for possible reactions at the electrode surface(s). For both compositions of Bim4S, a maximum current appeared to be reached before 5 V; this was especially visible in the derivative plot (Figure S4) where the current reaches a maximum before 5 V in the first oxidation step. Because of the widely different responses between Bim3S and Bim4S in the CVs, it was not certain which of these additives would be best for improving the cycling capabilities of PCL-PTMC with NMC, as a high current only in the first cycle for Bim4S-containing cells could be due to the formation of a passivating layer that improves the electrochemical stability in the long-term cycling, just as the low current for every cycle in the Bim3S cell also can be an indication of high electrochemical stability.

This example shows that the shortcomings of methods such as CV include difficulties in the analysis of complex results and a failure to take the sluggish kinetics of polymers into account. Because the potential in the cell is constantly changed during the measurement, the current response is dependent on the scan rate; with a scan rate that is too fast for a system with slow kinetics, any reactions will be driven to higher potentials than these would occur at if the potential was kept static. This also mimics the behavior in a battery cell poorly. For this purpose, several electrochemical methods have been developed with the aim to rectify the shortcomings of CV measurements.^45,46^ In this work, the staircase voltammetry (SV) was utilized as a method to more accurately show any reactions occurring at specific potentials. With a sufficient step time, the samples will reach a steady-state current, showing that the slow kinetics are overcome. Practical experience has shown that 1 h is sufficient for similar polymer electrolytes to appreciably approach equilibrium during the first steps where the materials should be electrochemically stable.

**Figure 4:**
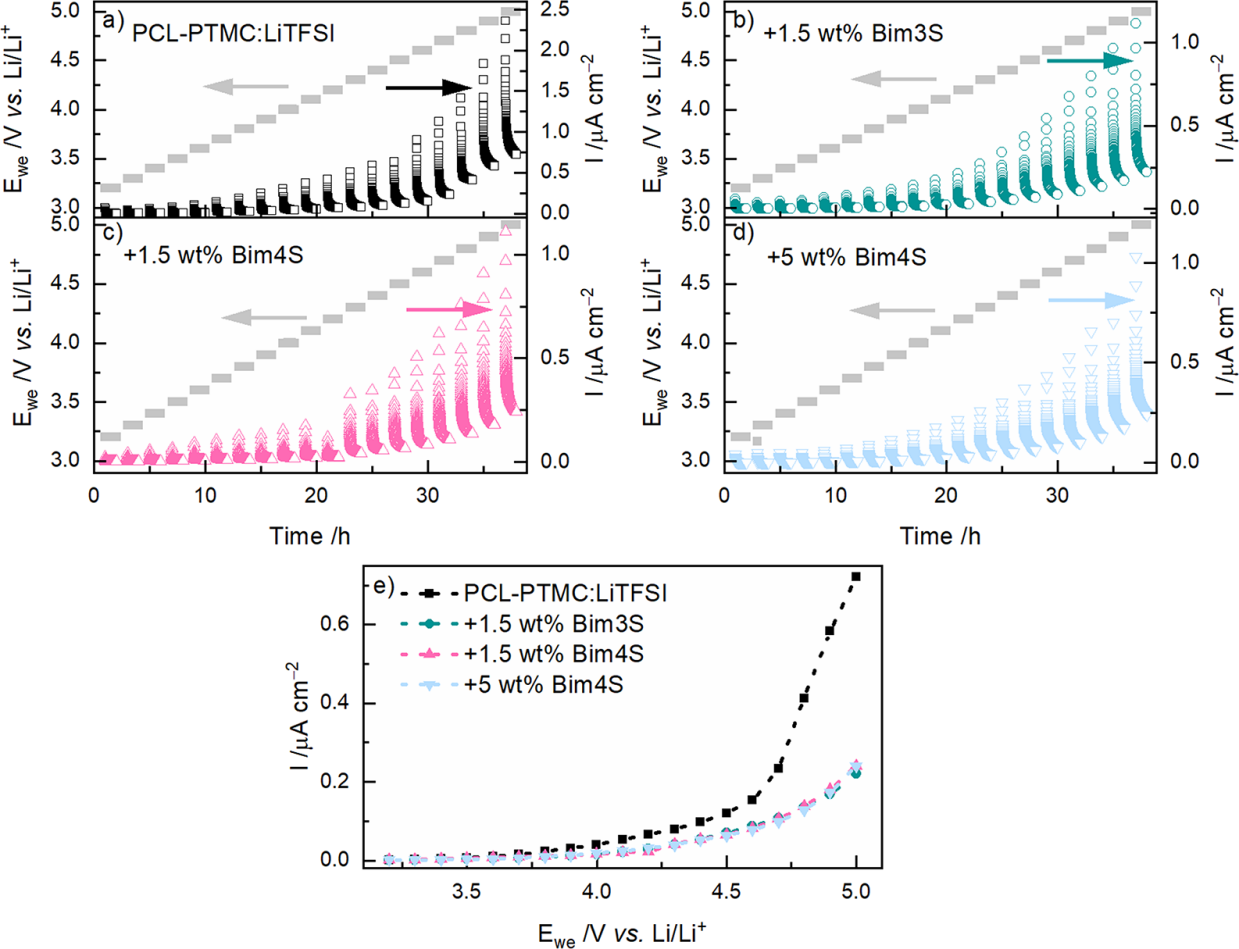
The current response during SV measurements for PCL-PTMC:LiTFSI (a) reference sample, (b) +1.5 wt % Bim3S, (c) +1.5 wt % Bim4S, and (d) +5 wt % Bim4S. (e) The final current for each potential step before removing the applied potential and allowing the cell to rest for 1 h. The measurements were performed at 40 °C.

The current response was measured for PCL-PTMC:LiTFSI and samples with zwitterionic additives, and the results are shown in Figure 4. Unlike the CV measurements, the results from SV showed that the reference sample had a higher current response than all zwitterion-containing SPEs and that both zwitterions had similar current response at all potentials and thus similar electrochemical stability.

With the SV method, any comparisons between different polymer hosts and SPEs with varied conductance due to, *e.g.*, thickness variations should be facilitated, as differences in ionic conductivity would be nullified with a long enough step duration. Since the ionic conductivity and *T*_g_ were largely similar (Figure 3), the variation in current response between the samples with and without zwitterions in Figure 4e can only be due to differences in the electrochemical stability of the electrolytes. During the first steps and at least until 3.5 V, the current is seen to stabilize for all samples, suggesting that a step duration of 1 h is sufficient. However, at later steps in the measurement, when the potential is above the electrochemical stability of the electrolyte, any formation of decomposition products is not only causing a change in current but is also seen to affect the resistance in the cell, and an equilibrium current is no longer reached. These measurements clearly show that both the onset and the extent of faradaic reactions were reduced with zwitterionic additives, with a low current and similar current response for all samples with zwitterions even above 4.5 V, compared to the PCL-PTMC:LiTFSI sample that had an almost exponential increase in current starting at ∼4.5 V. Although these results differ from the results of the CV measurements, they both point toward an increased electrochemical stability when adding zwitterions. According to these results, this enhanced electrochemical stability should in principle be enough to enable cycling against NMC regardless of the chosen zwitterion.

### CICC Cycling with the NMC Electrode

The setup for CV and SV measurements, being performed with a stainless steel working electrode that is expected to be inert toward the SPE within the examined potential range, is not representative of the environment in an operational battery. The electrochemical stability in these previous measurements did not take the surface chemistry of an active cathode material into account and, while the result is in some sense a relevant indicator of electrochemical stability, is not directly applicable to the battery cell during cycling against, *e.g.*, NMC. For example, in many cases, SPEs show an electrochemical stability close to 5 V, or even higher, during LSV or CV measurements but are unable to cycle against NMC.^19,47^ With the addition of zwitterions, the current response was decreased in the SV measurements (Figure 4e), but to better investigate the effect of the zwitterionic additives during cycling, cutoff increase cell cycling (CICC) was applied. This method allows monitoring of the capacity and voltage during galvanostatic cycling of cells with relevant electrodes, which gives information about irreversible degradation reactions appearing as the upper cutoff voltage is gradually increased during cycling.^32^

**Figure 5:**
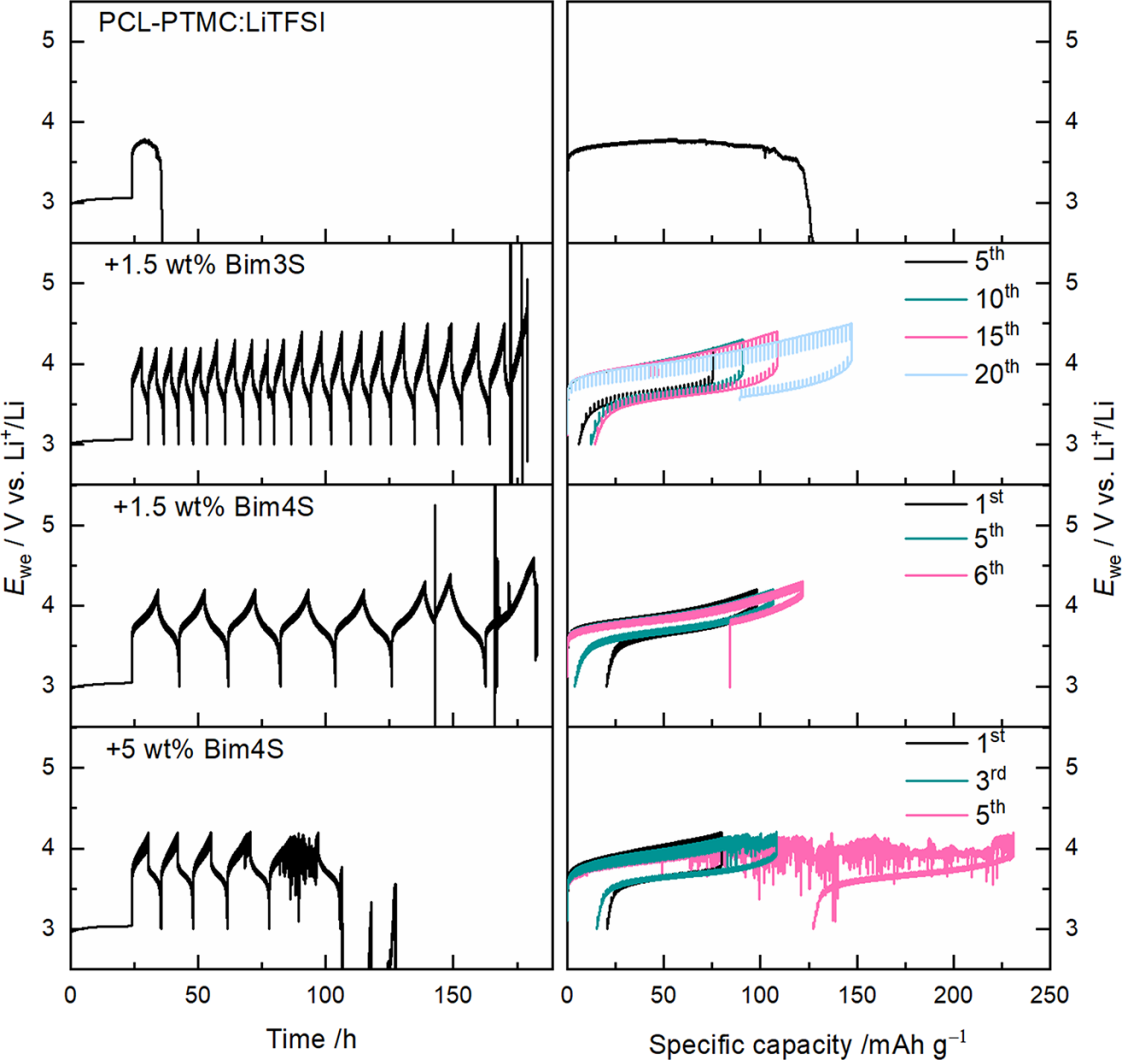
CICC measurements of PCL-PTMC:LiTFSI, reference and with zwitterionic additives. The left column shows the cycling profiles, and the right column shows the specific capacity for cycles of interest. Cycled with a current density of 0.025 mA cm^–2^.

In CICC measurements, in addition to analyzing the cycling profiles, the Coulombic efficiency is used to give an indication as to when the cell is failing, as seen in Figure S5, as opposed to only looking at the specific capacity for each cycle. Here, the reversibility of any reactions can be assessed through the Coulombic efficiency. Five cycles were performed at each voltage cutoff, and an unstable Coulombic efficiency at a specific voltage was taken as an indication of side reactions occurring in the cell. For the battery cells analyzed herein, the cycling profiles were sufficient for determining the potential at which failure occurred (see Figure 5).

For the reference cell, the common failure behavior when attempting to cycle a solid polymer electrolyte against NMC is shown. During the first, and only, charge, the cell fails to reach the upper cutoff, and a lot of the capacity gained is obtained during the arbitrary voltage noise behavior that occurs before failure. This type of voltage noise can be sustained by the cell for hundreds of hours if the mechanical properties of the solid polymer electrolyte allow it, but for this particular cell, it abruptly ended after 35 h due to a short circuit or Faradaic decomposition of the electrolyte.

As can also be seen in Figure 5, when zwitterions were added, the cells showed an increased cycling capability against NMC, with the 1.5 wt % Bim3S cell reaching an upper cutoff of 4.4 V, and failed once the cutoff was increased to 4.5 V, as is clear by the unstable Coulombic efficiency in Figure S5 for this composition. Following this, the battery cell showed unstable cycling until the upper cutoff was changed to 4.6 V; at this point, the cell was unable to cycle properly.

Both cells with Bim4S showed failure during the first five cycles when the upper cutoff potential was set to 4.2 V. The cell with 1.5 wt % Bim4S finished the first five cycles but failed to cycle properly once the cutoff was changed to 4.3 V.

ICI analysis was performed in conjunction with the CICC measurement to follow changes in the internal cell resistance during cycling (see Figure S6). Since the SPEs had similar thickness, the resistance values were normalized to the thickness of each SPE. For the cells that failed within the initial six cycles, a decrease in the resistance is seen. This could be due to an improved contact between the polymer electrolyte and the electrode or the breakdown of a passivating layer that was formed on the electrodes during the initial contact between SPE and electrodes, which if destroyed during cycling would decrease the resistance in the cell. A reduction of the electrolyte thickness would also result in a decrease in the resistance, but at the employed temperature and duration of the experiment, this would not be expected. It can be noted that all cells with the zwitterionic additives have a notably larger resistance compared to the reference cell, suggesting that the resistance is due to the presence of the zwitterions. The resistance in the cell containing 1.5 wt % Bim4S was smaller compared to that of the cell with 5 wt % Bim4S, which is apparent from the cycling profiles, which allowed the extraction of more capacity from the NMC-111 electrode. For the cell with 5 wt % Bim4S, the oxidative degradation reaction becomes an obvious failure mechanism during the fifth cycle.

The cell containing 1.5 wt % Bim3S showed a monotonic increase in resistance during cycling, and while it is not drastic, the increase in resistance is higher during the charging step compared to during the discharging step. For the different concentrations and types of zwitterions, the resistances in the cells do not match with the results from the EIS measurements in Figure 3a, which show that all samples have similar ionic conductivity. This could be a further indication that the zwitterionic additives form a passivating film on the NMC electrode, and this is what enabled the cells to perform more than one charge–discharge cycle with the NMC-111 electrode. From the CICC measurements, it is apparent that the zwitterionic additives improve the cycling behavior of PCL-PTMC:LiTFSI against NMC.

It could be noted that a number of research papers have been published showing a connection between the thickness of an SPE and its ability to cycle against NMC and other high-voltage cathodes, where a thicker SPE is less likely to exhibit the typical voltage noise failure mechanism.^38,48^ However, since the SPEs used for the CICC analysis all had similar thickness, 280 ± 30 μm, this sort of reasoning can be excluded from the analysis, and any favorable properties are instead most likely due to the zwitterionic additives.

Generally, when cycled with a liquid electrolyte, the upper cutoff voltage of NMC-111 is set to 4.2 V; at this potential, the NMC structure is expected to be stable. There is some increase in resistance, as seen by the ICI pauses in Figure 5, during the charging step that could be related to the structure instability of NMC at higher potentials.^49^

### *Ex Situ* XPS Measurements

With the measurements presented so far, there is an indication that there is some sort of degradation in the SPE in contact with NMC, which to some extent can be prevented with the zwitterionic additives. Thus, XPS was employed to analyze the chemical composition of the interface between the electrode and the polymer. This was done using galvanostatically cycled cells with NMC cathodes and SPEs, without and with zwitterions.

To examine the cells with XPS, the PCL-PTMC copolymer itself could not be utilized since the stickiness of the electrolyte would cause them to adhere too well to the electrodes, causing them to be peeled off and broken during disassembly. The corresponding SPE based on the PCL homopolymer, *i.e.*, PCL:LiTFSI, is semicrystalline and therefore considerably less sticky, and was utilized instead to render disassembly of cycled cells possible. The PCL-PTMC copolymer contains 80 mol % PCL, and therefore, the PCL SPE is expected to have a similar electrochemical stability behavior and thus be an acceptable alternative when performing postmortem analysis such as XPS measurements. Moreover, in the SV measurements (Figure S7), when using both PCL and PCL-PTMC as host material, there is a decrease in the current response when the zwitterionic additives are used compared to the reference samples with only LiTFSI. This shows that PCL can be used as a model material to analyze the effects of these zwitterionic additives, with strong implications also for the PCL-PTMC system. While the PCL:LiTFSI cell had a more erratic cycling behavior than the corresponding PCL-PTMC:LiTFSI cell during the SV measurements, it also showed an improvement in cycling when the Bim4S zwitterion was added to the SPE, just like in the PCL-PTMC:LiTFSI system. Due to a lower solubility of zwitterions in PCL:LiTFSI, only 1.5 wt % Bim3S or Bim4S was analyzed with XPS.

**Figure 6:**
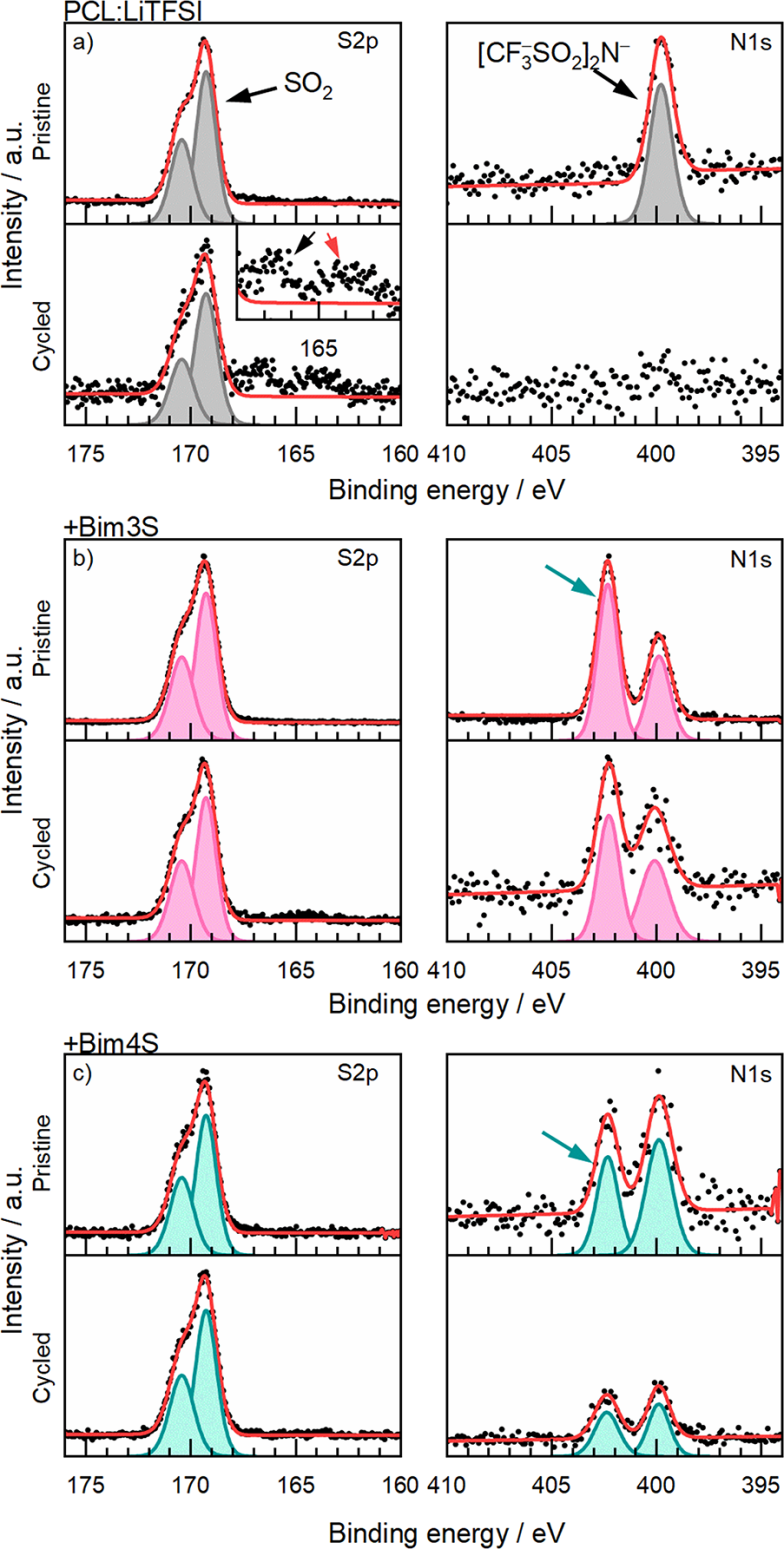
Postmortem XPS spectra, S2p and N1s, for the polymer side adjacent to NMC-111 for (a) PCL:LiTFSI, (b) +1.5 wt % Bim3S, and (c) + 1.5 wt % Bim4S. The SO_2_ salt peak in S2p, marked with an arrow in PCL:LiTFSI, was used for energy calibration. The inset for PCL:LiTFSI after cycling shows a black arrow highlighting the Li_*x*_S_*y*_O_*z*_ signal and a red arrow highlighting Li_2_S. The TFSI anion signal is marked with an arrow in the N1s spectrum of PCL:LiTFSI. Green arrows indicate peaks that are assumed to originate from the zwitterionic additives. The data have been normalized.

Without modifications, the PCL:LiTFSI SPE was not suitable for high-performance cycling, at least not at ambient temperature. To see how the zwitterions affected the polymer host, cells were cycled galvanostatically at a current density of 1 × 10^–6^ A cm^–2^ for five charge–discharge cycles (Figure S8), and then the cells were disassembled in their discharged state. After five cycles, all three cells, with and without zwitterions, had a somewhat stable Coulombic efficiency of around 80% (Figure S9). The XPS analysis (see Figure 6) was performed on the SPE side adjacent to the NMC-111, cycled and pristine (which had not been in contact with NMC). Due to overlaps in many of the signals and using a commercial NMC-111 cathode where the cathode particles likely have a coating to improve their performance and stability, the XPS analysis of the cathode is excluded from this work.

The SPE without zwitterions showed peaks of Li_*x*_S_*y*_O_*z*_ and Li_2_S forming after cycling, which are degradation products of the LiTFSI salt.^50^ Moreover, the signals from LiTFSI were reduced after cycling relative to the intensity of the peak signal in the pristine sample, indicating decomposition of the salt. In the S2p spectra of the zwitterion-containing SPEs, on the other hand, there were fewer signs of salt decomposition after cycling, and the spectra of the SPE with Bim4S were almost identical before and after cycling.

Similarly, in the N1s spectra, there was a significant difference between the SPEs without and with zwitterionic additives, especially after cycling. The salt peak, which was the only visible peak in the pristine PCL:LiTFSI N1s spectrum, disappeared after cycling, most likely due to the decomposition of the anion. This was again not the case when zwitterions were added; while the peaks decreased in intensity after cycling, they were still present. The SPEs with zwitterions displayed one additional peak that was assumed to be the nitrogens in the imidazolium of the zwitterion molecule. Between the two zwitterions, Bim3S seemed to have a better ability to prevent degradation based on the lesser degree of decrease in peak intensity before and after cycling, which is in agreement with the CV and CICC measurements.

We therefore posit that the zwitterions prevented the decomposition of the LiTFSI salt at the cathode surface during cycling, as Li_*x*_S_*y*_O_*z*_ and Li_2_S degradation was detected on PCL:LiTFSI but not in the SPEs with zwitterions (see the inset in Figure 6a). If the salt is completely consumed by degradation reactions in the vicinity of the electrode, it would be an explanation as to why the N1s peak is gone, or decreases, after cycling and why it is difficult to cycle such SPEs with NMC cathodes. A possible explanation for the disappearance of the peak is that the degradation products of the salt are gaseous, that they are contained within the bulk of the SPE and therefore not visible in the measurement, or that they are covered by the decomposition products of the polymer. In a study featuring the effect of zwitterions in an ionic liquid, a reduction in the anion concentration and the thickness of the SEI was noted when zwitterions were added, and it was concluded that the zwitterions prevented the anion from reaching the cathode, thus preventing its degradation.^51^ The phenomenon was further supported in a study utilizing *in situ* infrared–visible sum frequency generation spectroscopy on a tetraglyme-based electrolyte containing zwitterions.^39^ The XPS data presented in this study indicate that a similar interaction is in place, leading to less TFSI degradation.

### Galvanostatic Cycling

**Figure 7:**
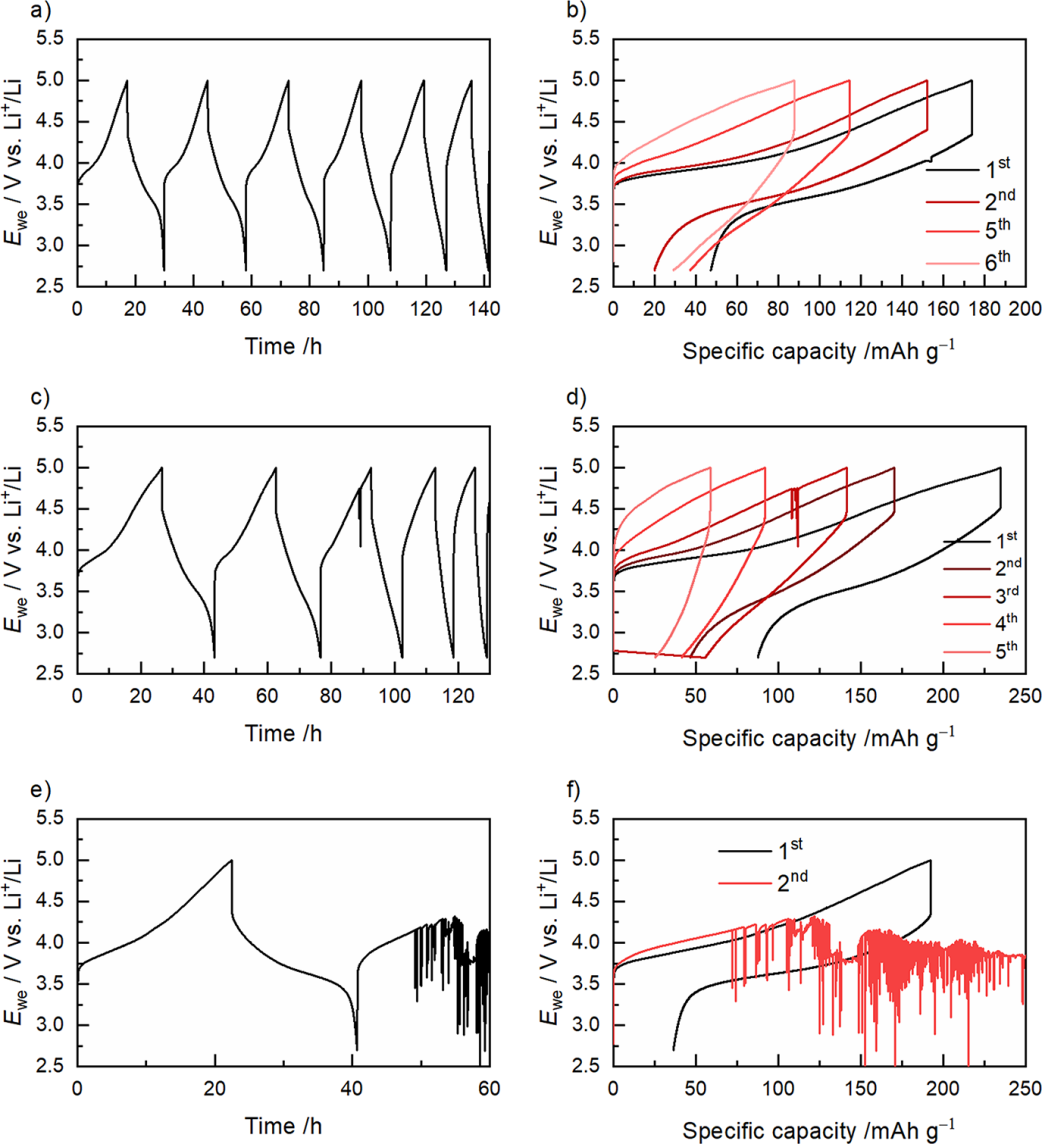
Cycling of cells containing NMC-111 and zwitterionic additives. PCL-PTMC:LiTFSI with (a, b) +1.5 wt % Bim3S, (c, d) +1.5 wt % Bim4S, and (e, f) +5 wt % Bim3S. The cells were cycled at 40 °C with a current density of 0.025 mA cm^–2^.

Finally, galvanostatic cycling was performed on cells containing the zwitterionic additives (see Figure 7). Both cells with 1.5 wt % zwitterion were capable of cycling more than five cycles, albeit with a clear increase in resistance following each cycle. Of the two zwitterions, the resistance increases with a higher rate in the cell with Bim4S, suggesting that Bim3S is the better of the two zwitterions.

The cell with 5 wt % Bim4S only completed one full cycle before displaying voltage noise behavior, but for the cells with a lower amount of additive, this erratic behavior was successfully prevented. For the NMC material, there is no clearly defined redox plateau; instead, the average de/lithiation potential is at 3.7 V vs Li^+^/Li.^52^ It is also around this potential that the voltage noise behavior is seen for cells that are failing. During normal galvanostatic cycling conditions, this is seen in the reference sample (Figure 1) and in the cell with 5 wt % Bim4S (Figure 7e–f). This suggests that there is a connection between the failure of the cell and the onset of redox reactions with the cathode material, which could be a degradation reaction of either the polymer or the LiTFSI salt, which is catalyzed by the transition metals on the surface of the NMC particles.

Both the galvanostatic cycling and the CICC data point toward an increased stability against NMC when using the zwitterionic additives. This improvement is significant, and it should also be considered that the thermal properties were not affected negatively and the ionic conductivity was even somewhat improved with the additives. Furthermore, considering that the reference cell presented in Figure 1 is a rare cell that was able to ″successfully″ complete one full cycle, in comparison to several cells with zwitterions being able to cycle for more than one cycle before showing signs of cell failure (shown in Figures 5 and 7), it is clear that the zwitterions introduce a positive effect. Because all test methods point to an increase in electrochemical stability when zwitterions were introduced, increasing the stability by additives appears as a viable strategy that is also easy to incorporate with SPEs.

While the Bim4S zwitterion had a higher solubility in the investigated polymer system, the Bim3S zwitterion showed better properties as an additive to improve battery cycling. CV measurements showed that the Bim3S zwitterion had an overall lower current response, and this proved to be an indication for the following cycling experiments. In the CICC measurements, Bim3S improved the cycling significantly compared to both the reference without zwitterions and the Bim4S zwitterion regardless of concentration, allowing the cell to reach an upper cutoff of 4.4 V, and during the galvanostatic cycling, the cell containing Bim3S had less issues with resistance increase. For both cells containing 1.5 wt % zwitterion, the increase in resistance was the main cause of the rapid decrease in capacity and thus the reason why the cells failed to cycle ″properly″.

Although the cycling is still limited, these results are promising and represent a clear step toward enabling the cycling of SPEs against cathodes such as NMC using this electrolyte material. Since the failure behavior observed when the cells contain both SPE and NMC as active materials points toward a chemical incompatibility between these two materials, this could potentially be chemically resolved by tailoring the interface and/or inhibiting the relevant side reactions. It is here shown that such problems are to some extent alleviated with the addition of zwitterions.

## Conclusions

In the current study, two zwitterionic additives were added to PCL-PTMC:LiTFSI in an attempt to increase the electrochemical stability enough to enable galvanostatic cycling against NMC-111. Several electrochemical methods, including CV, SV, and CICC, showed that the electrochemical stability was improved with all zwitterionic additives, indicating a stability limit considerably above the operating voltage for NMC.

The CICC tests conducted utilizing NMC as the working electrode showed that the inclusion of the Bim3S zwitterion resulted in improved electrochemical stability compared to the Bim4S zwitterion. Through the use of XPS on cycled cells, this improvement was correlated with a notable reduction in the decomposition of the TFSI anions when zwitterions were added to the SPE. This improvement facilitated markedly improved cycling of battery cells and improved the electrochemical stability of the electrolytes with NMC cathodes with SPEs containing the zwitterionic additives. Together, the results indicate that the issues are due to an incompatibility between the SPE and NMC rather than the electrochemical stability of the SPE, which was improved according to all employed techniques in this paper. While long-term cycling was still not feasible, the cycling performance of all zwitterion-containing SPEs was significantly improved, and with further optimization, long-term cycling could be made possible.

## Supporting Information Available

Photograph of SPEs; table of VFT fitting parameters; CV of cycles 1–5 and the differential plots of CV; Coulombic efficiency and average resistance during cycling of CICC measurements; SV of PCL; voltage profiles of PCL|NMC; and CE of PCL|NMC (PDF)
