# Supplementary material for: Improving the Electrochemical Stability of a Polyester–Polycarbonate Solid Polymer Electrolyte by Zwitterionic Additives
Source: ACS Appl Energy Mater. 2022 Jul 19;5(8):10002–12. doi: 10.1021/acsaem.2c01641 (PMC9400021; doi:10.1021/acsaem.2c01641)
Supplement: Supplementary file 1 — ae2c01641_si_001.pdf [file ae2c01641_si_001.pdf]

Supporting Information

Improving the electrochemical stability of polyester–polycarbonate solid polymer electrolyte by zwitterionic additives

Isabell L. Johansson[a], Christofer Sångeland[a], Tamao Uemiya[b], Fumito Iwasaki[b], Masahiro Yoshizawa-Fujita[b], Daniel Brandell[a], Jonas Mindemark[a,]*

[a] Department of Chemistry – Ångström Laboratory, Uppsala University, Box 538, SE-751 21 Uppsala, Sweden

[b] Department of Materials and Life Sciences, Sophia University, 7-1 Kioi-cho, Chiyoda-ku, Tokyo 102-8554, Japan

* Corresponding author. Email: jonas.mindemark@kemi.uu.se

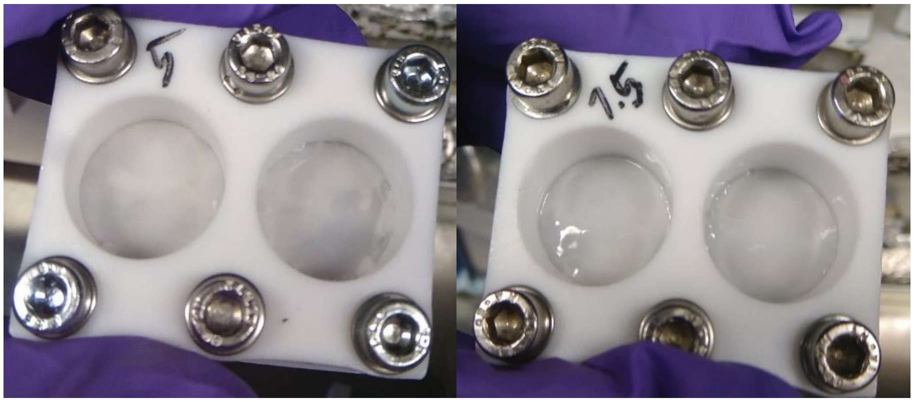

Figure S1. Photo showing solvent-cast films of PCL-PTMC:LiTFSI (Left) +5 wt% Bim3S and (Right) +1.5 wt% Bim3S. There was a loss in glossiness due to precipitation when too much zwitterion was added.

Table S1. Fitting parameters obtained from fitting conductivity data to the Vogel–Fulcher–Tamman equation, $S = A \times (1000/T)^{-1/2} exp\left(\frac{-B}{(1000/T - T_0)}\right)$. Values here have some uncertainty as there were no duplicate sample measurements.

**Composition**	***A* / S cm^{-1} K$^{1/2}$**	***B* / K**	***T_0* / K**	***B*/*T_0***
**PCL-PTMC:LiTFSI**	10.9	1796.2	153.5	11.7
**PCL-PTMC:LiTFSI + 1.5 wt% Bim3S**	1.9	1380.8	162.4	8.5
**PCL-PTMC:LiTFSI + 1.5 wt% Bim4S**	33.5	2013.1	139.5	14.4
**PCL-PTMC:LiTFSI + 5 wt% Bim4S**	0.1	562.3	214.2	2.6

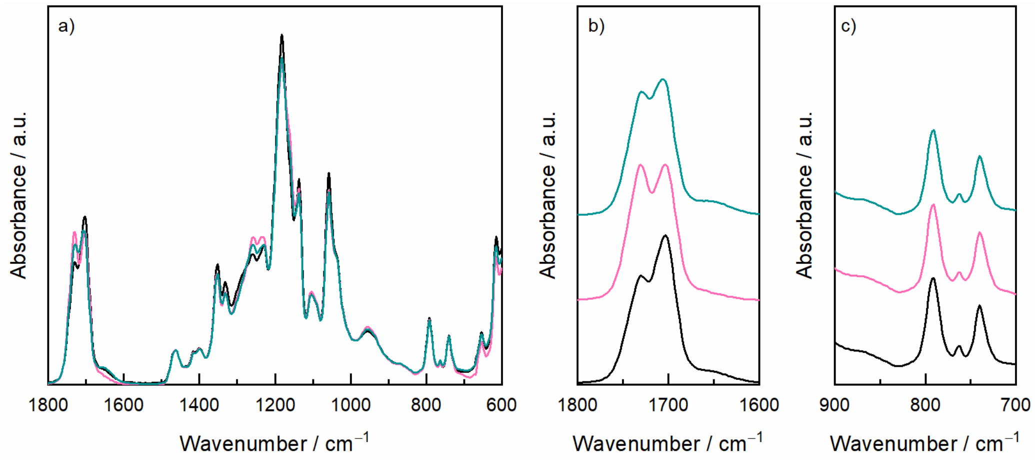

Figure S2. FTIR spectra of (black) PCL-PTMC:LiTFSI, (pink) + 1.5 wt% Bim3S, and (green) + 1.5 wt% Bim4S, in the a) 1800–600 cm^{-1}, b) 1800–1600 cm^{-1}, and c) 900–700 cm^{-1} region.

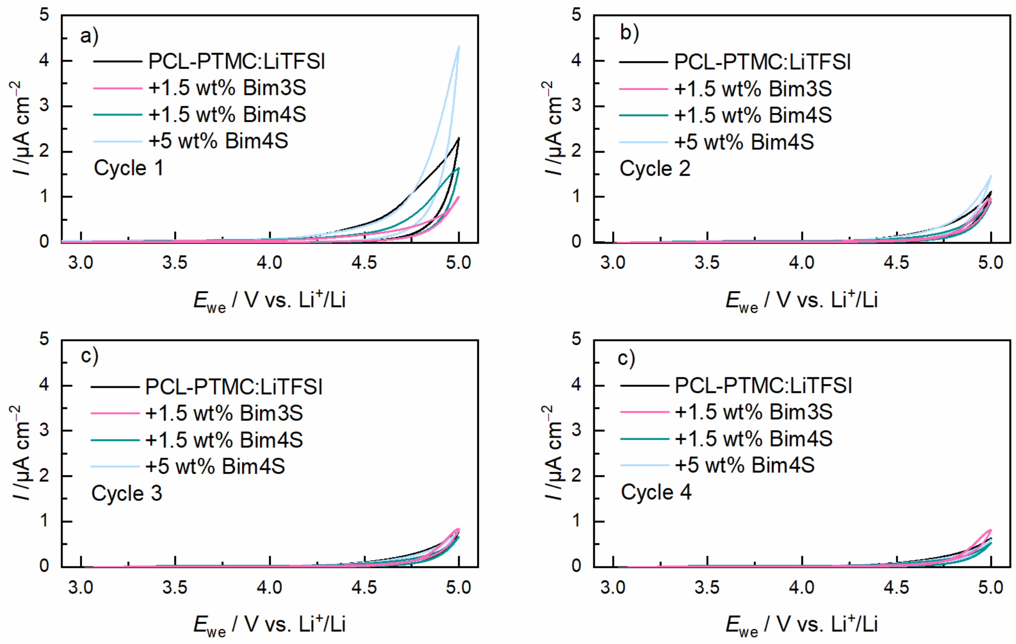

Figure S3. Cyclic voltammograms of PCL-PTMC:LiTFSI with and without zwitterionic additives, separated by a) cycle 1, b) cycle 2, c) cycle 3, and d) cycle 4.

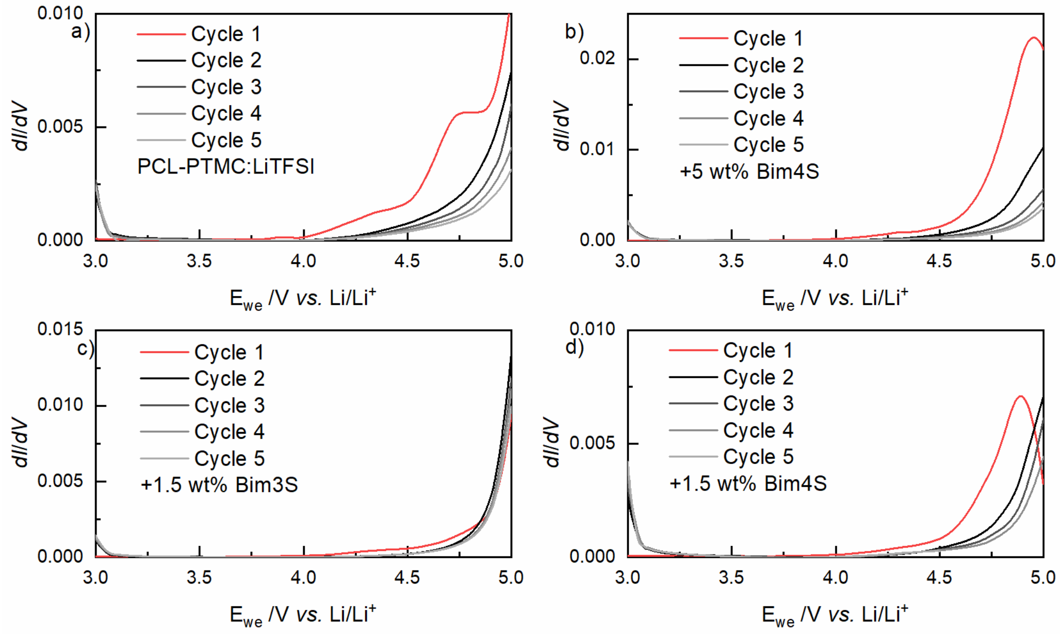

Figure S4. The differential of the LSV data, grouped by sample composition a) PCL-PTMC:LiTFSI, b) +1.5 wt% Bim3S, c) +1.5 wt% Bim4S, and d) +5 wt% Bim4S. LOESS smoothing has been applied to the differential data.

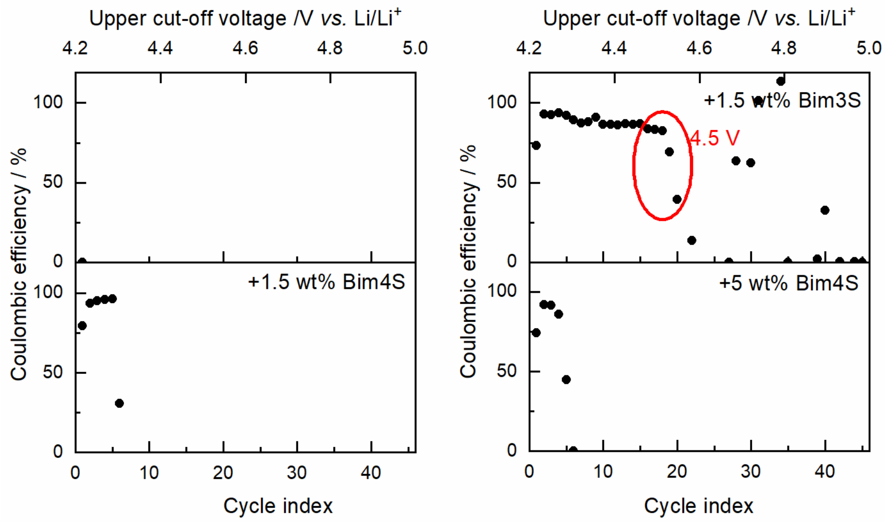

Figure S5. The Coulombic efficiency of Li|PCL-PTMC|NMC-111 cells during CICC measurements. The potential at which failure occurred is marked in red for the cell containing 1.5 wt% Bim3S; all other cells failed to cycle within the first six cycles.

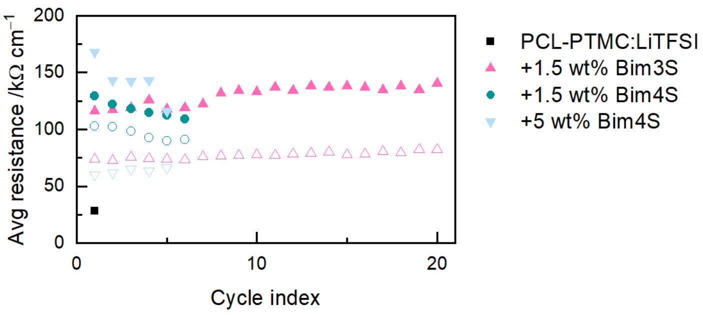

Figure S6. Internal average cell resistance, during charge (filled symbols) and discharge (hollow symbols), from ICI analysis during the CICC measurements of a cell with the reference electrolyte, and of cells containing SPEs with the zwitterionic additives. The average resistance was divided with the thickness (280±30 µm) of each solid polymer electrolyte in order to normalize the resistance and compare the cells. Only the 20 first cycles are shown for PCL-PTMC:LiTFSI+1.5 wt% Bim3S.

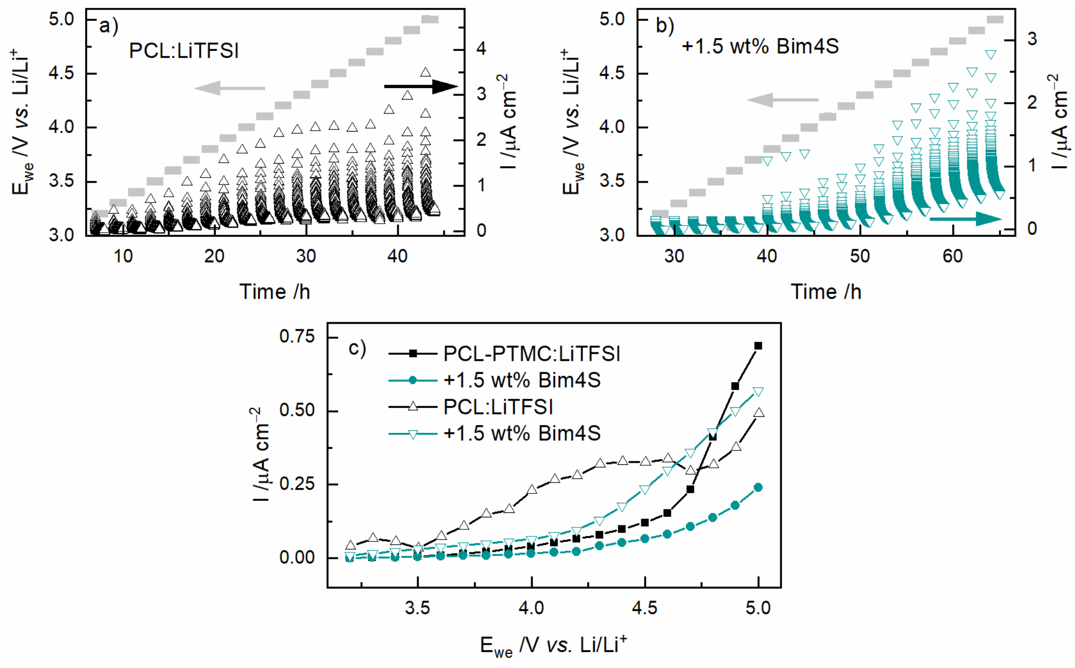

Figure S7. Current response during SV measurements for a) PCL:LiTFSI and b) +1.5 wt% Bim4S. The time before starting the measurement was varied to allow a stable OCV. Measurements were performed at 80 °C. The final current for each potential step during SV measurement, before removing the applied potential and allowing the cell to rest for 1 h. c) A comparison of the response in current between PCL-PTMC:LiTFSI (40 °C), shown as filled symbols, and PCL:LiTFSI (80 °C) with and without 1.5 wt% Bim4S, shown as open symbols.

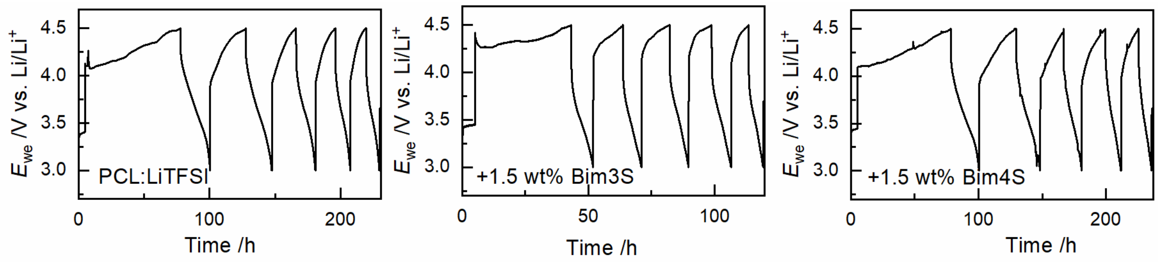

Figure S8. Voltage profiles of cycling PCL:LiTFSI-based SPEs, with and without zwitterionic additives, with NMC-111 for XPS.

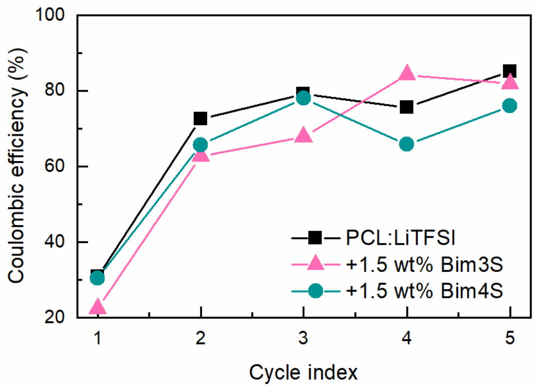

Figure S9. Coulombic efficiency of cells while cycling PCL:LiTFSI-based SPEs with NMC-111 at a current density of 1 μA cm^{-2}.
